# Concurrent Relations between Face Scanning and Language: A Cross-Syndrome Infant Study

**DOI:** 10.1371/journal.pone.0139319

**Published:** 2015-10-01

**Authors:** Dean D’Souza, Hana D’Souza, Mark H. Johnson, Annette Karmiloff-Smith

**Affiliations:** 1 Centre for Brain and Cognitive Development, Department of Psychological Sciences, Birkbeck, University of London, London, United Kingdom; 2 Department of Experimental Psychology, University of Oxford, Oxford, United Kingdom; University of Leuven, BELGIUM

## Abstract

Typically developing (TD) infants enhance their learning of spoken language by observing speakers’ mouth movements. Given the fact that word learning is seriously delayed in most children with neurodevelopmental disorders, we hypothesized that this delay partly results from differences in visual face scanning, e.g., focusing attention away from the mouth. To test this hypothesis, we used an eye tracker to measure visual attention in 95 infants and toddlers with Down syndrome (DS), fragile X syndrome (FXS), and Williams syndrome (WS), and compared their data to 25 chronological- and mental-age matched 16-month-old TD controls. We presented participants with two talking faces (one on each side of the screen) and a sound (/ga/). One face (the congruent face) mouthed the syllable that the participants could hear (i.e., /ga/), while the other face (the incongruent face) mouthed a different syllable (/ba/) from the one they could hear. As expected, we found that TD children with a relatively large vocabulary made more fixations to the mouth region of the incongruent face than elsewhere. However, toddlers with FXS or WS who had a relatively large receptive vocabulary made more fixations to the *eyes* (rather than the mouth) of the incongruent face. In DS, by contrast, fixations to the speaker’s overall face (rather than to her eyes or mouth) predicted vocabulary size. These findings suggest that, at some point in development, different processes or strategies relating to visual attention are involved in language acquisition in DS, FXS, and WS. This knowledge may help further explain why language is delayed in children with neurodevelopmental disorders. It also raises the possibility that syndrome-specific interventions should include an early focus on efficient face-scanning behaviour.

## Introduction

From birth, infants are exposed to a socially rich environment, frequently seeing and hearing talking faces. Moreover, early on they show biases that orient them towards socially-relevant information such as face-like stimuli [1–2] and speech-like sounds [3–5]. Through their massive experience with talking faces, infants develop the capacity to detect face-voice synchrony [6–10], to match lip movements to speech sounds [11–13], and to use visual speech cues to enhance their speech perception [14–18]. Moreover, they learn to use visual speech cues even in the absence of auditory speech information. For instance, when presented with silent video clips of speakers reciting sentences, 4- and 6-month-olds can visually discriminate their native language from an unfamiliar one [19].

Although much research has focused on the infant’s ability to process auditory speech [20], and while some has examined how infants extract visual information from talking faces [19] and integrate auditory/visual speech input (e.g., [14]), little is known about how these basic-level abilities are used to construct higher-level language skills. This is because few studies have explored the link between face scanning and language ability. One study, involving typically developing (TD) infants and infants at high risk of developing autism, used growth curve analyses to demonstrate that 6-month-olds who fixated more on their mother’s mouth during live mother-child interaction develop language at significantly higher rates and have significantly higher expressive scores at 24 months than infants who fixated more on their mother’s eyes [21]. This effect amounted to a difference of more than 4 months in developmental age, which suggests that gaze to mouth is a useful predictor of individual differences in language development. Importantly, no differences were found between the high-risk infants and TD controls. The finding was also independent of autism diagnosis at outcome. Thus, the authors concluded that their finding reveals a *normative* mechanism of language development. Because an infant’s ability to perceive phonemes facilitates his or her acquisition of language [22], Young and colleagues [21] hypothesise that any developmental phenomenon—including visual attention to the mouth—that facilitates speech perception will also facilitate later language development. Furthermore, adult research by Schwartz, Berthommier, and Savariaux [23] has shown that viewing a speaker’s mouth improves the intelligibility of their speech when embedded in noise. In other words, not only does visual input from the mouth contribute to infants’ learning of auditory phoneme categories [24]—and thus to their ability to acquire language—but it may be especially helpful at all ages for speech perception under noisy conditions [25].

The adult findings of Schwartz et al. [23] have yet to be demonstrated in TD children. However, the findings of Young et al. [21] have received support from an event-related potentials (ERP) study. Key, Stone, and Williams [26] presented 9-month-old infants with photographs of an unfamiliar face. On 30% of the trials, they replaced the eyes or mouth of the face with the corresponding parts (eyes, mouth) of a different face. Whereas eye changes only had an effect on face perception mechanisms (as reflected by a larger occipito-temporal N290 ERP component with short latency), the N290 response to mouth changes was also correlated with a parent-report measure of receptive communication. In other words, the size of the N290 brain response to visual changes in the mouth region of a face was positively correlated with language ability in a group of TD 9-month-olds. Thus, observing lip movements provides speech-related information and plays an important role in communication (see also [27–29]).

Conversely, Reid and Striano [30] suggest that focusing on the *eyes* (i.e., rather than the mouth) is crucial for language development, because eye gaze is a precursor to complex joint attention skills, imitation, and acquiring new knowledge and skills (see also [31]). For example, children acquire expressive labels for objects partly by following another person’s gaze to an object while that person provides the corresponding label. Indeed, in line with Reid and Striano’s prediction, greater attention to eyes in 6-month-olds has been associated with better joint attention skills at 8 and 12 months [32], and gaze following in 6-month-olds has been positively associated with vocabulary size at 18 months [33–34].

Why is there disagreement over whether the eyes or the mouth are more important as the focus of attention for language development? We suggest that the importance of mouth gaze versus eye gaze may change as a function of developmental time. According to the literature, eye gaze is important for joint attention, triadic attention, emotional face processing, imitation, etc., but mouth gaze may play a critical role in extracting visual information (e.g., lip reading) that facilitates understanding of unfamiliar, noisy, or confusing (auditory) speech. Indeed, the infant’s focus of attention to features in talking faces changes over the course of the first year of life. Lewkowicz and Hansen-Tift [35] presented 4-, 6-, 8-, 10-, and 12-month-old English-learners with video clips of a female speaking either the infants’ native (English) or a non-native (Spanish) language. Irrespective of language familiarity, 4-month-olds looked longer at the eyes, 6-month-olds looked equally long at the eyes and the mouth, and 8- and 9-month-olds looked longer at the mouth area. Twelve-month-olds looked equally long at the eyes and mouth when native speech was spoken, whereas they maintained their looking towards the mouth area for the non-native language. The authors argue that expertise (e.g., perceptual narrowing) explains this behaviour: non-native speech requires complementary audio-visual cues from the mouth region. Although Lewkowicz and Hansen-Tift did not measure language ability in the studied children, they concluded that—from around 8 months of age—attention to a speaker’s mouth “corresponds to the emergence of speech production during typical development and, thus, suggests that access to the redundant audiovisual cues available in the mouth is critical for normal development” (p.1435).

From this literature, we hypothesized that face-scanning patterns would be associated with language ability and, in particular, would change over developmental time. Such findings could have important implications for atypical development. Children with neurodevelopmental disorders often present with language delay [36]. Could their language delay be partly explained by atypical face scanning patterns? Interestingly, there is some evidence that visual scanning patterns differ across atypical populations [37]. For instance, individuals with Williams syndrome (a rare genetic disorder) spend significantly more time looking at the eyes of a face than TD controls [38–41], whereas those with fragile X syndrome (a different, more common genetic disorder) often avoid eye contact altogether [42]. (To our knowledge, the relationship between eye gaze aversion in FXS and language ability has never hitherto been explored.)

To address our question (‘Could language delay be partly explained by atypical face scanning?’), in the present study we decided to investigate the face-scanning patterns of infants and toddlers with different neurodevelopmental disorders (Down syndrome [DS], fragile X syndrome [FXS], Williams syndrome [WS]; see Table 1). These particular disorders were selected because their pathology (aetiology, pathogenesis, morphologic changes, clinical manifestations) is well defined; other disorders, e.g., autism, are multifactorial syndromes, with many causes, many subtypes, and no clear unifying mechanisms at either the molecular, cellular, or systems level. Our aim was to ascertain whether face-scanning patterns relate to language ability–and if so, *how* they relate to it.

**Table 1:** A brief description of three neurodevelopmental disorders.

Group	Description	References
Down syndrome (DS)	The most common chromosome abnormality, DS is caused by the trisomy of chromosome 21. It is characterised by mild to moderate intellectual disability, decelerated maturation (neoteny), and a number of other physical, cognitive, and behavioural atypicalities, including poor verbal working memory and language delay.	[43–44]
Fragile X syndrome (FXS)	The most common form of inherited intellectual disability in males, FXS is caused by an expansion of the unstable CGG repeat within the FMR1 gene. Affected individuals often present with an attentional deficit, anxiety, gaze aversion, and language delay.	[45–46]
Williams syndrome (WS)	A rare genetic disorder, caused by a hemizygotic microdeletion of approximately 1.6 Mb containing ~28 genes on chromosome 7 (7q11.23). WS is characterized by an uneven cognitive profile with particularly weak visuo-spatial construction abilities. Affected individuals are often hyper-social and attracted to faces. Although language ability is a relative strength in later development, they present with language delay in early development.	[47–48]

We presented participants with two talking faces (one on each side of a screen) and a sound (/ga/). One face (the congruent face) mouthed the syllable that the participants could hear (/ga/), while the other face (the incongruent face) mouthed a different syllable (/ba/) from the one they could hear (/ga/). If mouth gaze does indeed play a role in extracting visual information that facilitates understanding of unfamiliar or confusing (auditory) speech, then we should expect that the children who focus their attention towards the mouth of the incongruent face will have relatively larger vocabularies than those who direct their attention elsewhere.

Based on the results of Lewkowicz and Hansen-Tift [35] and others [21, 23, 26], we predicted that the 16-month-old TD controls would focus more on the incongruent than congruent face and that those whose focus was more on the mouth region of the incongruent speaking face would have a larger vocabulary. We also predicted no correlation between gaze towards the eyes and vocabulary size. This is because, though eye gaze is important for cognitive and social development, it is not relevant for children in this particular experimental context. That is, although children may show a general bias towards the eyes, the eyes provide no useful information in this context. So, unlike gaze to the mouth, there should be no relationship between gaze to the eyes and vocabulary size. Finally, we hypothesized that infants and toddlers with WS would fixate for longer on the eyes (as the previous literature suggests [38]) than the other groups, and hence obtain less visual cue information from the mouth. In other words, we predicted that they would not use visual mouth cues. Given the current state of the literature, no a priori predictions could be made with respect to the DS and FXS groups. Although we expected toddlers with FXS to avoid fixating on the eyes, it was impossible to predict whether they would focus on the visual mouth cues or avoid the face altogether (see Table 2 for a summary of the predictions).

**Table 2:** Summary of predictions.

Group	Prediction	Reason
Typical development (TD)	TD children who more often fixate on the mouth in the incongruent face will have larger vocabularies than those who fixate elsewhere.	Mouth movements provide visual information that may facilitate understanding [14–18].
Down syndrome (DS)	No a priori predictions were made.	
Fragile X syndrome (FXS)	No specific predictions were made. However, it was expected that the toddlers with FXS would not fixate on the eyes of either face.	Gaze aversion is a characteristic of FXS [42].
Williams syndrome (WS)	Children with WS will fixate on the eyes of both faces. They will make few fixations to the mouth of either face, and thus no relationship between mouth gaze and vocabulary will be detected.	Individuals with WS are drawn to eyes [38].

## Methods

### Participants

A total of 95 infants (around 15 months of age) and toddlers (around 30 months of age) from the three neurodevelopmental disorders were tested: 22 infants and 21 toddlers with DS, 14 toddlers with FXS (too few infants being available for testing), and 12 infants and 26 toddlers with WS. It was not possible to match infants with FXS on chronological age, because FXS is often diagnosed later in development. The mean age of diagnosis is around 3 years for boys with FXS and 3.5 years for girls with FXS [49]. The participants had been clinically diagnosed and/or genetically tested respectively for full trisomy 21, mutation of the FMR1 gene, or deletion of the ELN gene. Data collected from these children were compared with data from 25 typically developing (TD) controls. Data from these controls were made available through the British Autism Study of Infant Siblings (BASIS, www.basisnetwork.org; NHS NRES London REC 08/H0718/76) who had been tested with the same materials and procedure. These TD controls did not have a sibling with autism. We did not include data from children at risk of developing autism. For all participants included in the present study, we verified that the primary language spoken in the home was English, even though a few of the children were exposed to more than one language. The race and ethnicity of the participants reflected the race and ethnicity of the general population. For instance, in each group the majority of participants (over 75%) were “White” or “White British”, while fewer than 10% were “non-White” and fewer than 15% were “British Mixed”.

Because children with DS, FXS, or WS have a mental age (MA) of approximately half their chronological age (CA), data from the TD control group were compared with data from MA-matched groups as well as CA-matched groups. Participants’ mental ages were obtained for the purpose of comparison, using the Mullen Scales of Infant Learning (MSEL [50]). Data from one participant with WS were excluded from all analyses, because the 37-month-old had a (relatively) high MA (31.25 months) that was 2.76 standard deviations (SD) above the group mean.

Table 3 displays mean CA for each CA-matched group (*TD controls*, *DS infants*, *WS infants*). A one-way ANOVA shows that the CA-matched groups did not significantly differ on CA, *F*_2,56_ = 1.60, *p* = .212. Because the distribution of CA data in the WS and TD control groups looked slightly bimodal, an Independent-samples Kruskal-Wallis test was also carried out to confirm the results of the ANOVA. This non-parametric test also yielded no significant difference between the groups on CA, *H*(2) = 1.32, *p* = .516.

**Table 3:** Mean chronological age (CA) and mental age (MA) for each group.

Group	*N*	CA in months *(SD)*	MA in months *(SD)*
TD controls	25	15.48 *(0*.*82)*	16.60 *(2*.*50)*
DS infants^a^	22	16.23 *(1*.*81)*	8.46 *(2*.*45)*
WS infants^a^	12	16.13 *(2*.*00)*	8.71 *(1*.*89)*
DS toddlers^b^	21	28.83 *(6*.*86)*	15.86 *(4*.*52)*
FXS toddlers^b^	14	34.39 *(8*.*32)*	15.34 *(4*.*42)*
WS toddlers^b^	25^c^	30.14 *(8*.*23)*	16.11 *(4*.*53)*

^a^ These two groups were CA-matched to the TD control group

^b^ These three groups were MA-matched to the TD control group

^c^ This table does not include the participant who was excluded from the analyses for having a relatively high MA (see main text).

MA data were normally distributed. A one-way ANOVA revealed that MA did not differ significantly across the MA-matched groups (*TD controls*, *DS toddlers*, *FXS toddlers*, *WS toddlers*), *F*_3,80_ = 0.32, *p* = .809 (Table 3).

### Ethics Statement

The study was explained to participants’ caregivers beforehand, and written consent from them on behalf of their infants/toddlers was obtained. All experimental procedures were in accordance with the Declaration of Helsinki (www.wma.net/en/30publications/10policies/b3/), and were approved by the ethics committees of the Department of Psychological Sciences (Birkbeck, University of London), and the National Research Ethics Service (UK Health Research Authority).

### Design

**Fig 1:**
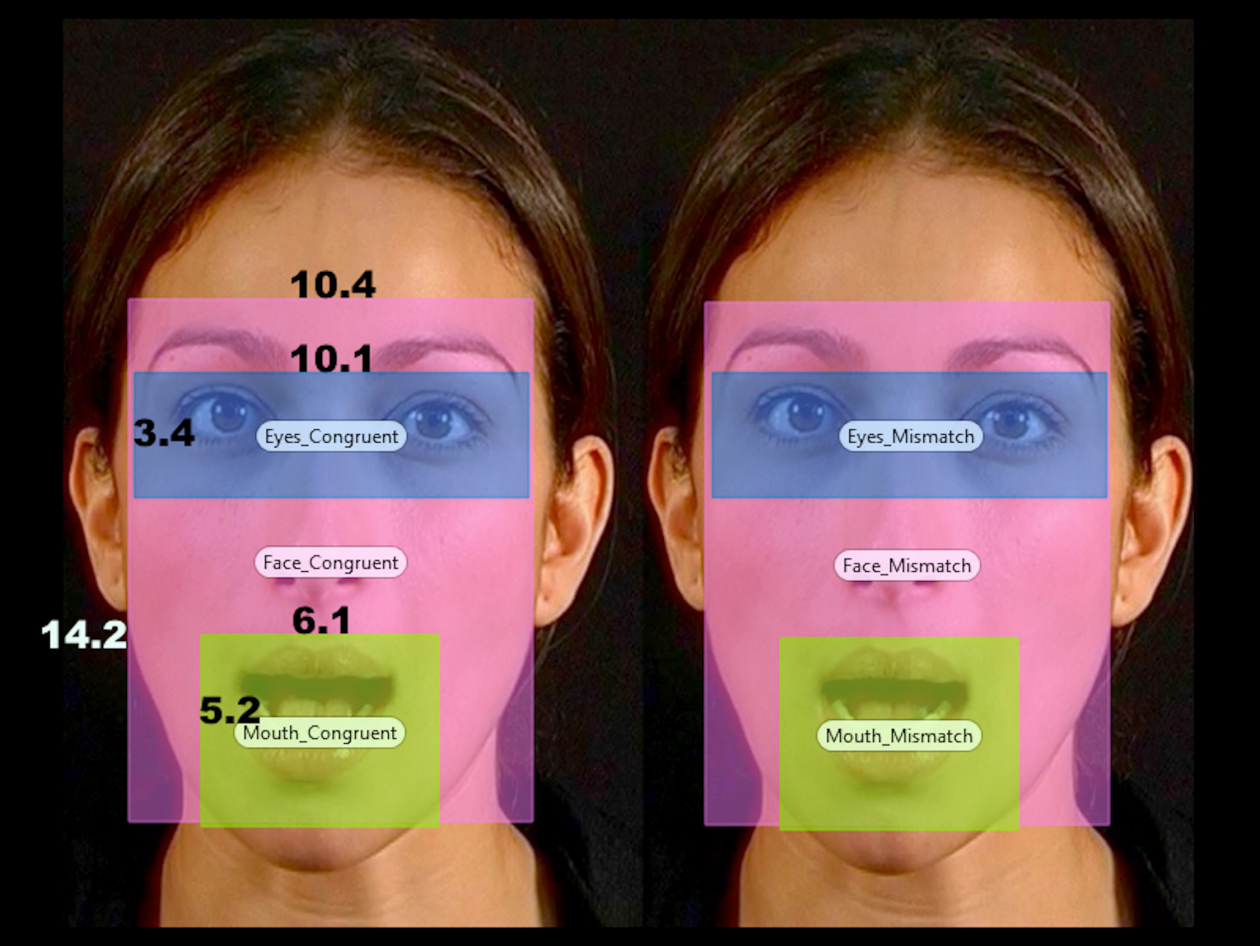
An example of the visual stimuli used in the experiment, including the positioning and sizes in visual angle of the Areas-Of-Interest (eyes, face, and mouth).

The design was adapted from [14] and [51]. Two adult female faces, with moving lips, were presented side-by-side on a screen (Fig 1), with loudspeakers placed behind it. There were two trials. In each trial, the participants were presented with two talking faces (one on each side of the screen) and a sound. In one trial, the left-hand face mouthed the syllable /ba/ and the right-hand face mouthed the syllable /ga/, while the sound /ga/ was simultaneously heard. In the other trial, the left-hand face mouthed the syllable /ga/ and the right-hand face mouthed the syllable /ba/, while the sound /ga/ was heard throughout. Thus, in each trial, one speaking face was *congruent* (i.e., the visual stimulus matched the auditory stimulus) and the other speaking face was *incongruent* (i.e., there was a mismatch between the visual stimulus and the auditory stimulus). The two trials (i.e., the position of the faces) were counterbalanced. There were two other conditions, in which the sound /ba/ (rather than /ga/) was heard. Thus, all participants took part in four counterbalanced conditions in total. However, the visual /ga/ and the auditory /ba/ produce an illusory percept (the McGurk effect) that will be reported elsewhere.

### Materials

The same stimuli were used as in Kushnerenko et al. ([14]; see also [51–52]). The stimuli consisted of two 12-second video clips of two speaking faces, side-by-side, of the same female native English speaker articulating either the /ba/ or /ga/ syllable against a black background (see Fig 1 for an example of the visual stimuli, including the positioning and sizes in visual angle of the Areas-of-Interest). In one video clip, the left-hand face articulated the /ba/ syllable, while the right-hand face articulated the /ga/ syllable. In the other video clip, the left-hand face articulated the /ga/ syllable, while the right-hand face articulated the /ba/ syllable. In both video clips, the sound /ga/ was played. The faces were approximately life size. The video clips and stereo soundtracks were digitised at a rate of 25 frames per second and 44.1 kHz with 16-bit resolution.

Each 12 s video clip started with lips fully closed, with the face being silent for the first nine frames (360 ms). The subsequent voiced section lasted for seven frames, followed by three frames of the mouth closing. Thus, sound onset began 360 ms after the onset of the two visual face stimuli, and the voiced section lasted for 280 ms. The mouth opening for the visual /ga/ stimulus started about 260 ms prior to sound onset. For the visual /ba/, it started simultaneously with sound onset, with the lips pressing together around 280 ms prior to sound onset. Total duration of the stimulus (i.e., one mouth movement and a simultaneous sound) was 760 ms, and stimulus onset asynchrony (SOA) was 760 ms, with each video clip (15 repetitions of mouth movements and a simultaneous sound) being 12 s long.

In other words, there were two video clips (two conditions). Each video clip lasted 12 seconds. Each video clip included 15 repetitions of mouth movements and a simultaneous sound (/ga/). Each repetition (which included the opening and closing of the mouth) lasted for 760 ms. Each repetition included the sound (/ga/), which lasted for 280 ms. Incongruent face stimuli were created by dubbing the auditory /ga/ onto the visual /ba/. The consonantal burst in the audio file was aligned with the consonantal burst in the video file.

A Tobii T120 remote eye tracker (Tobii Technology AB) was used to capture moment-to-moment point of gaze at a sampling rate of 120 Hz, with measurement accuracy of about 0.5°. The visual stimuli were presented on a 34 x 27 cm TFT liquid crystal display monitor, with a resolution of 1280 x 1024 pixels and a response rate of 4 ms. The tracking equipment and stimulus presentation were controlled using Tobii Studio 2.1.14. A camera mounted directly above the horizontal midpoint of the screen was used to monitor and record infant behaviour. Auditory stimuli were delivered via two speakers positioned behind the display monitor and facing the participant.

#### Measuring vocabulary levels

The MacArthur-Bates Communicative Developmental Inventory (*CDI* [53]) was used to assess language in the infants and toddlers. The CDI is a standardised parent report measure of vocabulary size (comprehension and production). It comes in two forms: *Gestures and words* and *Words and sentences*. The CDI *Gestures and words* scale covers the period from 8 to 16 months; the CDI *Words and sentences* scale from 16 to 30 months.

In the current study, parents were given the CDI *Gestures and words* scale. Although the chronological age of some of our participants was above that of the ceiling (16 months) of this younger scale of the CDI, language delay is common among those with neurodevelopmental disorders and, indeed, no atypical participant in our study was at ceiling. The word list consists of 396 words in 19 semantic categories. For each word, the questionnaire measures whether the child only understands the word or whether the child both understands and produces the word.

For the analyses, raw scores (i.e., the number of words the child understands and the number of words the child both understands and says) rather than standardized/normalized scores were used. This is because tests such as the CDI are only standardized for TD children and thus lack sensitivity at the extreme ends of the normal distribution. Yet infants and toddlers with neurodevelopmental disorders often score below the lowest percentile provided for the norms included in the CDI and similar tests.

### Procedure

The questionnaire was posted to parents prior to the day of testing and completed within two weeks of their visit to the laboratory (where the experimental task was carried out). The parents were instructed to mark down which words their child could understand and which words their child could both understand and say.

For the experimental task, the same procedure was used as in [14] and [52], i.e., infants sat on their parent’s lap, in a dimly lit featureless room, facing the stimulus-presentation screen, with their eyes at a distance of approximately 60 cm from the screen. The experimenter sat behind a curtain and observed the infant, using Tobii Studio LiveViewer via a camera that was positioned centrally above the screen. The infants’ eye movements were recorded using Tobii Studio 2.1.14. Caregivers were asked to close their eyes during the experiment. Calibration was carried out using 5 points: one in each corner of the screen and one in the centre of the screen. Before each trial, a colour animation and interesting sounds were played to attract the infant’s attention to the centre of the screen. Once the child’s attention was focused on the screen centre, the attention grabber was terminated and the trial was started simultaneously. The total duration of the experimental procedure did not exceed 10 minutes.

### Analysis

For each participant, the quality of recording was measured as a percentage (the number of eye tracking samples that were correctly identified, divided by the number of attempts, so 50% means that one eye was found for the full recording or that both eyes were found for half the time. The eyes cannot be detected when a participant is looking away from the screen; this will result in a lower percentage). The quality of recording was at least 25% in all participants. The quality of recordings did not significantly differ across CA- or MA-matched groups, *H*(2) = 4.76, *p* = .093, *F*_3,81_ = 0.90, *p* = .448, respectively (see Table 4). A non-parametric test was used for the CA-comparison because the data in the WS infant group had a continuous (rather than a normal) distribution.

**Table 4:** Quality of data for each group.

Group	*N*	Mean (%)	*SD*
TD controls	25	82.2	12.7
DS infants^a^	22	80.1	14.3
WS infants^a^	12	63.4	25.1
DS toddlers^b^	21	75.3	13.1
FXS toddlers^b^	14	80.1	12.8
WS toddlers^b^	25^c^	77.4	19.4

^a^ These two groups were CA-matched to the TD control group

^b^ These three groups were MA-matched to the TD control group

Areas-Of-Interest (AOIs) were delineated around the eyes and mouth (Fig 1). These were defined before data were collected [51–52]. Duration of fixations and fixation count were calculated off-line using Tobii Studio and Tobii fixation filter (Tobii Inc.). *Duration of fixations* is the total duration (in seconds) of all fixations within an AOI. We decided to look also at fixation count, because there is evidence that it is positively correlated with at least some aspects of language acquisition [54]. *Fixation count* is the number of times the participant fixated on an AOI. It is a useful measure of attention-holding mechanisms [55–58], because it tells us something about how a stimulus *maintains* infants’ attention when other (conflicting) stimuli are also vying to capture attention [56].

All data were tested for normality. Data values that were above or below 2 standard deviations from the group mean were judged *a priori* to be outliers, and hence removed from the analysis (unless the data were transformed–see below). This decision was based on the literature [59]. If the data were non-normal (i.e., if *Z*_*Skewness*_ > ±2 and Kolmogorov-Smirnov *p* < .05), then they were transformed (log 10, reflected log 10, or arcsine, as appropriate). If the transformed data were non-normal (e.g., bimodal), then the untransformed data were analysed using the appropriate non-parametric test (e.g., Mann-Whitney).

## Results

First, we decided to characterise looking patterns across the different groups. To do so, we analysed where on the face each group was looking. Specifically, for each AOI (eyes/mouth, congruent/incongruent) we analysed duration and number of fixations.

### CA-matched infant comparison (N.B. no infants with FXS participated)

#### Looking patterns–duration of fixations

**Fig 2:**
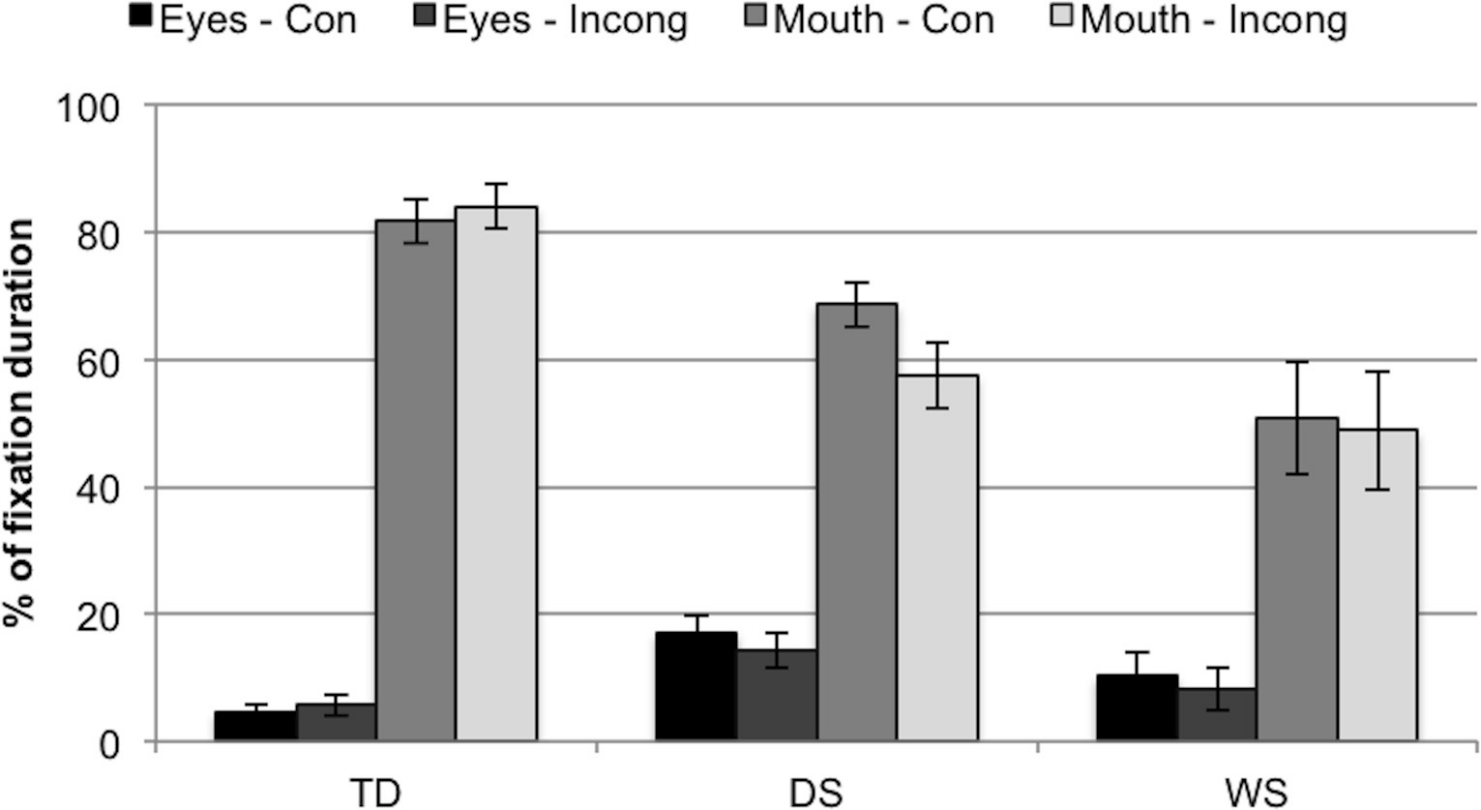
Fixation durations to eyes/mouth AOIs as a percentage of fixation durations to the face AOI, for each group (TD, DS, WS) and face (congruent, incongruent). Eyes-Con = eyes AOI in the congruent face. Eyes-Incong = eyes AOI in the incongruent face. Mouth-Con = mouth AOI in the congruent face. Mouth-Incong = mouth AOI in the incongruent face. TD controls fixated for longer at the mouth AOIs than infants with DS or WS (all, *p* < .01). Error bars represent one standard error of the mean.

Four fixation duration measures were taken: *eyes in the congruent face*, *eyes in the incongruent face*, *mouth in the congruent face*, and *mouth in the incongruent face*. These measures were re-coded as *percentages*, i.e., fixation durations to the eyes/mouth AOIs as a percentage of fixation durations to the face AOI, for each group (TD, DS, WS) and face (congruent, incongruent) (see Fig 2).

Because some of the data were positively skewed while some were negatively skewed (*Z*_Skewness_ > ±2), nonparametric tests were carried out. Friedman’s ANOVAs revealed significant differences between the four measures in each of the three groups, *χ* ^2^(3) = 54.04, *p* < .001 (TD), *χ* ^2^(3) = 42.61, *p* < .001 (DS), *χ* ^2^(3) = 17.00, *p* = .001 (WS). Wilcoxon tests were used to follow up this finding. Bonferroni corrections were applied. Therefore, all effects are reported at a .008 level of significance. TD controls fixated significantly more on the mouth (relative to the entire face) than on the eyes (relative to the entire face) (all, *p* < .001, *r* >-.76). No significant differences were found between the mouths in both faces, nor between the eyes in both faces (both, *p* > .906). The same pattern emerged in DS (i.e., for all comparisons *p* < .001, *r* >-.59, except for comparisons between both mouth AOIs [*p* = .391] and between both eyes AOIs [*p* = .540]). However, this pattern may be weaker in WS, because although the infants with WS fixated less on the eyes in the incongruent face than on the mouth in either face (both, *p* = .001, *r* = -.76), comparisons between the eyes in the congruent face and the mouth AOIs did not survive the Bonferroni correction (both, *p* = .030, *r* = -.48).

Furthermore, Kruskal-Wallis tests revealed significant differences between the groups in fixation percentages to the eyes in the congruent face (*H*(2) = 12.71, *p* = .002, but not in the incongruent face *p* = .066) and to the mouth in both the Congruent and Incongruent faces (*H*(2) = 12.02, *p* = .002, *H*(2) = 15.73, *p* < .001, respectively). These findings were followed up with pairwise comparisons. Bonferroni corrections were applied. Therefore, all effects are reported at a .017 level of significance. The TD controls looked significantly less at the eyes in the congruent face than the infants with DS, *Z* = -3.56, *p* < .001, *r* = .53. The TD controls also looked more at the mouth in the congruent and incongruent faces than the infants with DS (*Z* = 2.42, *p* = .016, *r* = -.39, *Z* = 3.36, *p* = .001, *r* = -.52, respectively) or WS (*Z* = 3.24, *p* = .001, *r* = -.49, *Z* = 3.30, *p* = .001, *r* = -.52, respectively).

In other words, TD controls fixated for longer at the mouth AOIs than infants with both forms of neurodevelopmental disabilities.

#### Looking patterns–number of fixations

**Fig 3:**
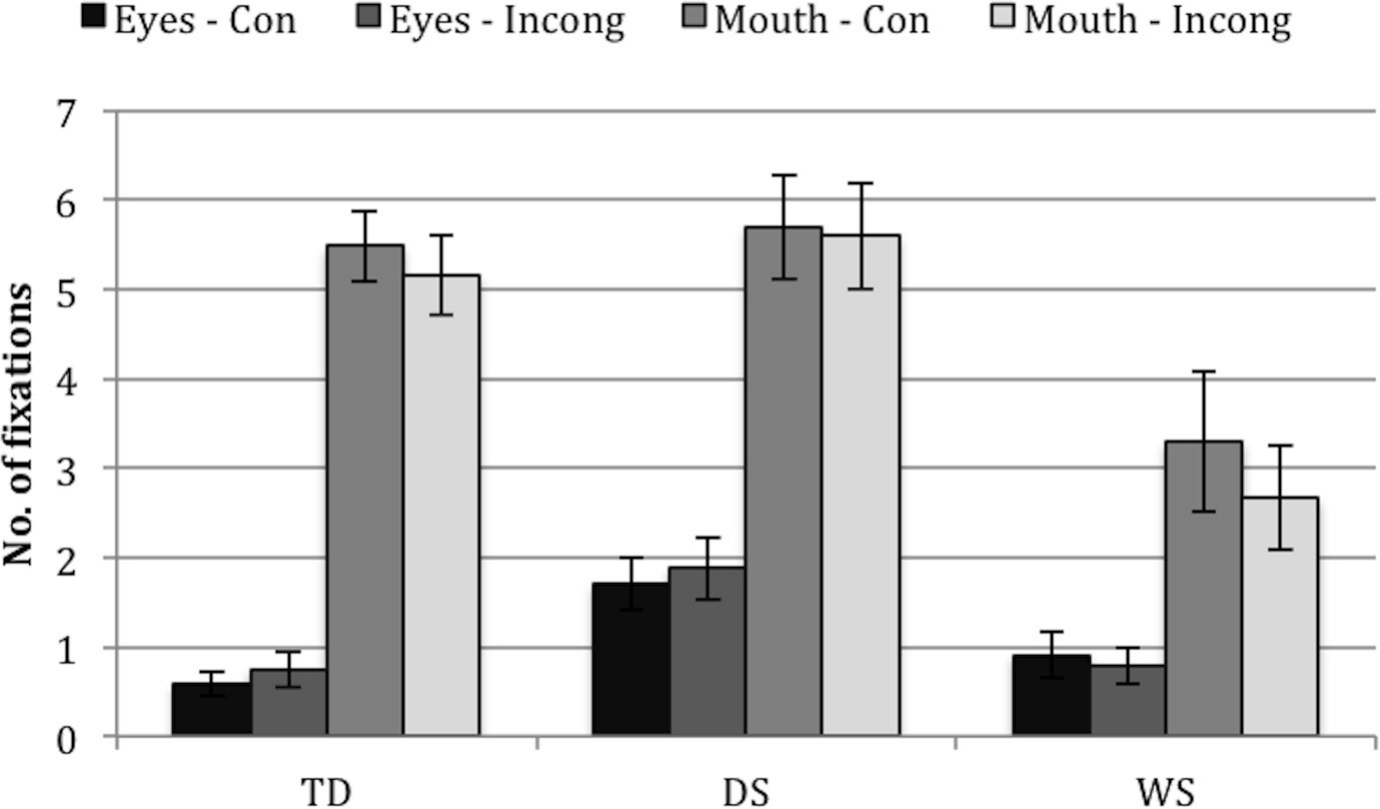
Number of fixations to eyes/mouth AOIs for each group (TD, DS, WS) and face (congruent, incongruent). Eyes-Con = eyes AOI in the congruent face. Eyes-Incong = eyes AOI in the incongruent face. Mouth-Con = mouth AOI in the congruent face. Mouth-Incong = mouth AOI in the incongruent face. Infants with WS made fewer fixations at the mouth AOIs than infants with DS or TD controls. Error bars represent one standard error of the mean.

To further characterise looking patterns in the three groups, we analysed number of fixations: *eyes in the congruent face*, *eyes in the incongruent face*, *mouth in the congruent face*, and *mouth in the incongruent face*. Fig 3 shows number of fixations to eyes/mouth AOIs for each group (TD, DS, WS) and face (congruent, incongruent).

Because some of the data were positively skewed (even when transformed, *Z*_Skewness_ > ±2), nonparametric tests were carried out on the (untransformed) data. Friedman’s ANOVAs revealed significant differences between the four measures in each of the three groups, *χ* ^2^(3) = 50.88, *p* < .001 (TD), *χ* ^2^(3) = 24.21, *p* < .001 (DS), *χ* ^2^(3) = 17.06, *p* = .001 (WS). Wilcoxon tests were used to follow up this finding. Bonferroni corrections were applied. Therefore, all effects are reported at a .008 level of significance. TD controls made significantly more fixations to the mouths than to the eyes (all, *p* < .001, *r* >-.59). No significant differences were found between the mouths in both faces, or between the eyes in both faces (both, *p* > .239). The same pattern was found in DS and WS (i.e., for all comparisons *p* < .01, *r* >-.77, except for comparisons between both mouth AOIs [both, *p* > .868] and between both eyes AOIs [both, *p* > .583]).

Furthermore, Kruskal-Wallis Tests revealed significant differences between the groups in number of fixations to the eyes in both the Congruent and Incongruent face (*H*(2) = 10.44, *p* = .005, *H*(2) = 7.20, *p* = .027, respectively), and to the mouth in both the Congruent and Incongruent face (*H*(2) = 6.79, *p* = .034, *H*(2) = 10.78, *p* = .005, respectively). These findings were followed up using pairwise comparisons. Bonferroni corrections were applied. Therefore, all effects are reported at a .017 level of significance. The TD controls made significantly fewer fixations at the eyes in the congruent and incongruent faces than the infants with DS, *Z* = -3.22, *p* = .001, *Z* = -2.60, *p* = .009. The TD controls also made more fixations at the mouth AOIs in the congruent and incongruent faces than the infants with WS (*Z* = 2.47, *p* = .014, *Z* = 2.81, *p* = .005, respectively). Infants with DS made more fixations to the mouth than infants with WS in the incongruent face (*Z* = 3.13, *p* = .002) but not in the congruent face *Z* = 2.24, *p* = .025 [this did not survive the Bonferroni correction) (see Fig 3).

In other words, infants with WS made fewer fixations to the mouth than both TD controls and infants with DS.

#### Predicting language

Analyses were carried out to ascertain whether the group differences in visual scanning had any relation with receptive or expressive language. Our sample was too small for us to run statistical tests such as ‘multiple regression’. However, our *N* was large enough for simple regressions. Because we predicted that looking at the *mouth* in the incongruent face would correlate with greater language ability, and because the TD infants looked more at the mouth than the eyes, we decided to ascertain whether it predicts language ability.

In TD controls, fixation duration to the mouth in the incongruent face (as a percentage of fixation durations to the entire incongruent face) predicted expressive language (*B* = 0.57, *SE B* = 0.26, β = .46, *p* = .038 [which explains 21% of the variance]) but not receptive language (*p* = .107). Conversely, number of fixations to the mouth in the incongruent face predicted receptive language (*B* = 8.68, *SE B* = 4.06, β = .42, *p* = .044, [17% of the variance]) but not expressive language (*p* = .850) in TD controls.

However, neither fixation duration nor number of fixations predicted receptive/expressive language in the two atypically developing groups (all, *p* = ns). But because these groups did not focus attention on the mouth AOIs to the same extent as the TD controls, and because they made a lot of fixations to the eyes, we decided to see whether fixations to the *eyes* predict language ability in these children. The results were surprising. Although fixation duration to the eyes in the incongruent face (as a percentage of fixation durations to the entire incongruent face) did not predict language ability in any group (all, *p* = ns), *number of fixations to the eyes* in the incongruent face predicted receptive language in infants with WS (*B* = -17.36, *SE B* = 5.53, β = -.79, *p* = .020 [which explains 62% of the variance]) but not in DS or TD controls (both, *p* = ns).

Nevertheless, number of fixations to the eyes did not predict expressive language in any of the groups (all, *p* = ns). Intrigued, we delved deeper and found that the measure does, however, predict both receptive and expressive language in WS (*B* = -1.83, *SE B* = 0.70, β = -.64, *p* = .026 [41% of the variance], *B* = -2.69, *SE B* = 0.95, β = -.67, *p* = .018 [44% of the variance], respectively), when language was assessed by the experimenters using the Mullen, a live test, rather than the CDI, a parental questionnaire. We reanalysed fixation duration percentage and found that it also predicted receptive and expressive language in WS as evaluated using the Mullen, *B* = -0.11, *SE B* = 0.05, β = -.63, *p* = .038 (40% of the variance), *B* = -0.20, *SE B* = 0.06, β = -.77, *p* = .006 (59% of the variance), respectively. This was not observed in the other two groups [all *p* = ns].)

Looking at the eyes in the incongruent face was thus important for the infants with WS, whereas it was the mouth region that was important for the TD controls. But did it matter what face (incongruent, congruent) the eyes or mouth were in? To explore this, we decided first to compare fixation counts (FC) at the eyes AOIs (i.e., FC_EYE_ = incongruent eyes/[incongruent eyes + congruent eyes]), and then FC at the mouth AOIs (FC_MOUTH_ = incongruent mouth/[incongruent mouth + congruent mouth]). Regression analyses were again used to ascertain whether FC_EYES_ or FC_MOUTH_ predict language ability in these children (for these final sets of analyses, we decided to focus on *receptive language* which, as mentioned earlier, was found to be dependent on fixation count in the TD controls).

#### Eyes

**Fig 4:**
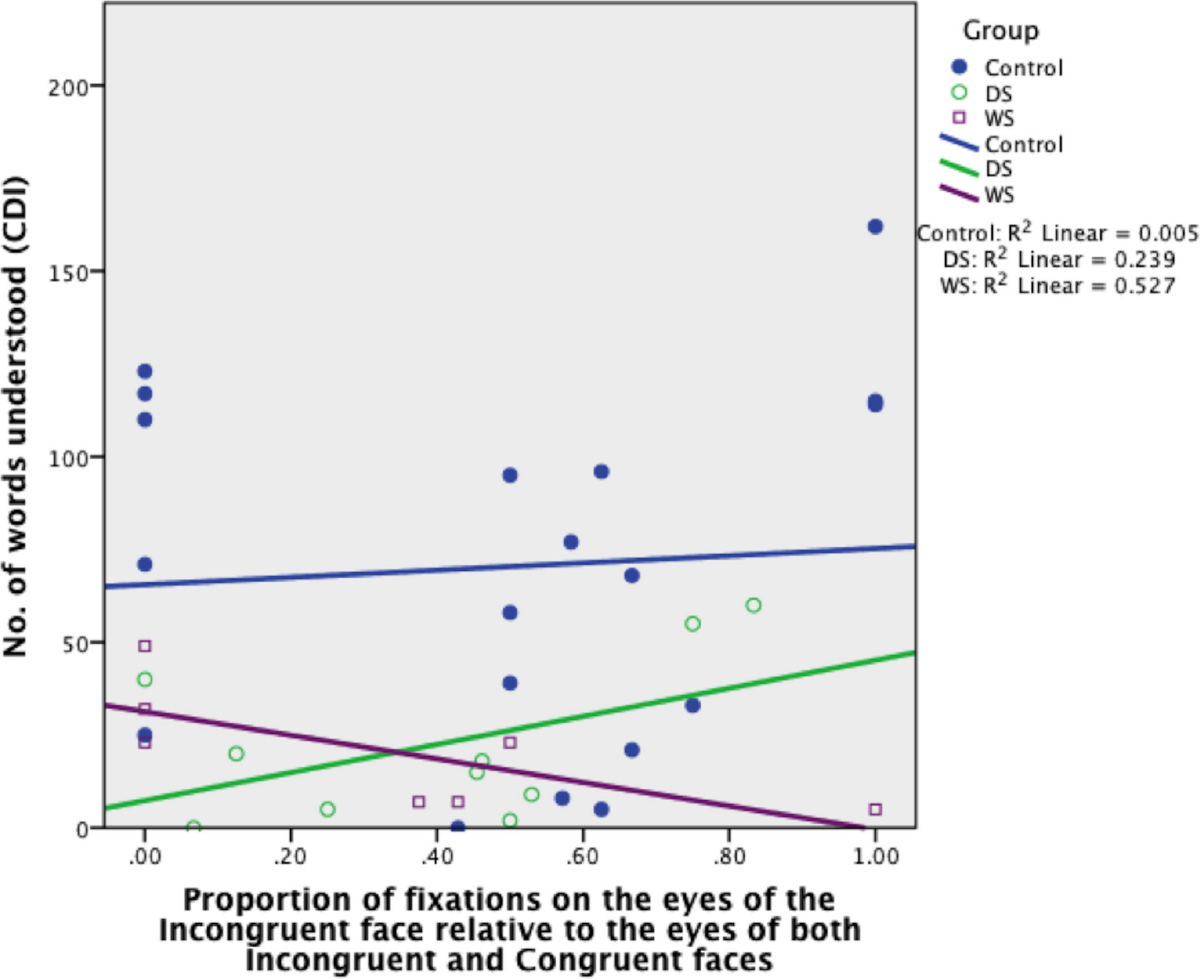
Proportion of fixations on the eyes of the Incongruent face relative to the eyes of both Incongruent and Congruent faces, organised by Group (TD control, DS, WS).

FC_EYES_ did not predict receptive language in any of the groups (all *p* > .05), although there was a trend in the WS group, *B* = -31.76, *SE B* = 13.45, *β* = -.73 (*R* ^2^ = .53, *p* = .065; see Fig 4).

#### Mouth

**Fig 5:**
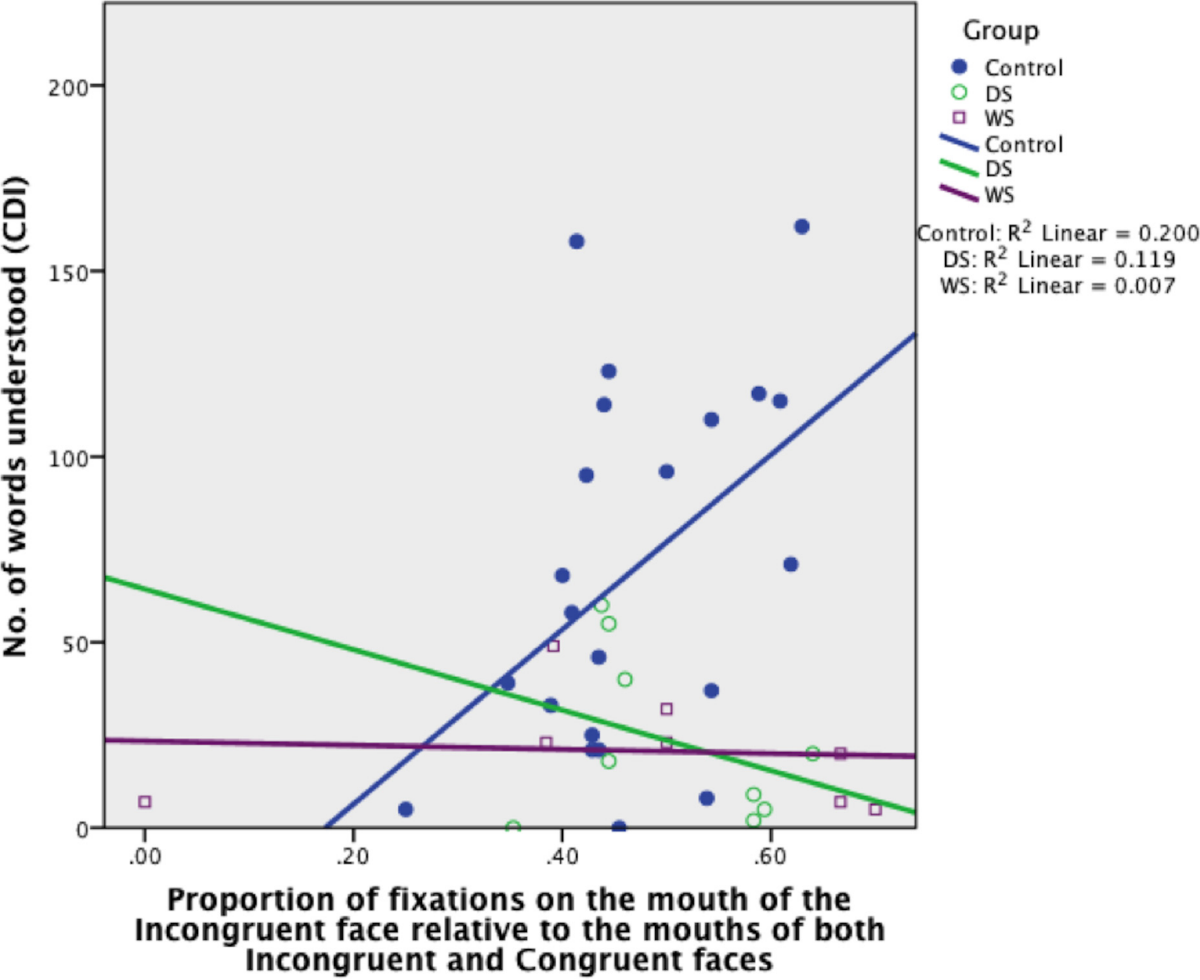
Proportion of fixations on the mouth of the Incongruent face relative to the mouths of both Incongruent and Congruent faces, organised by Group (TD control, DS, WS).

**Fig 6:**
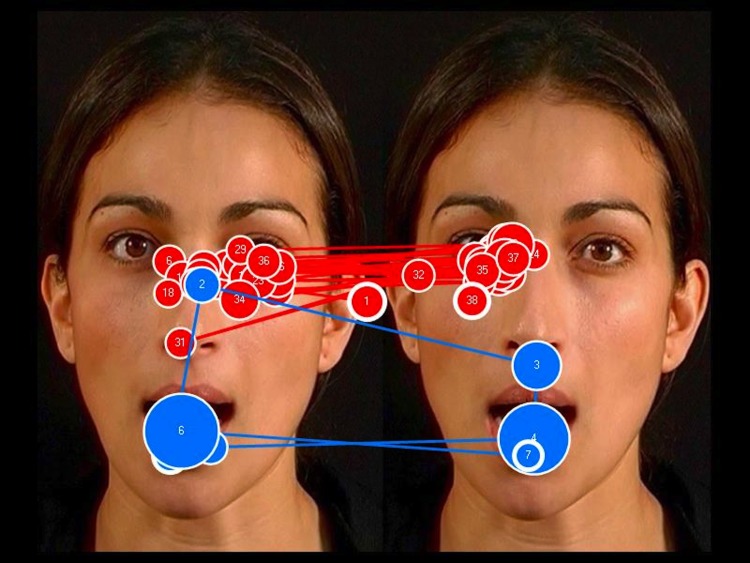
The face-scanning patterns of two typically developing (TD) children: the blue path represents the scanning pattern of a child with a receptive vocabulary of 213 words; the red path represents the scanning pattern of a child with a receptive vocabulary of 8 words. TD infants who focused on the incongruent mouth had a larger vocabulary than those who focused on the congruent mouth. The incongruent mouth is the one on the left.

FC_MOUTH_ was a significant predictor of receptive language and accounted for 20% of its variability in the TD control group, *B* = 235.23, *SE B* = 105.04, *β* = .45 (*R* ^2^ = .20, *p* = .037) (see Fig 5). This indicates that the more fixations to the mouth in the Incongruent face, relative to the mouths in both faces, the greater is the child’s receptive vocabulary (for every .1, the infant understands an extra 24 words on average; see Fig 6 for an example TD participant’s gaze pattern). By contrast, FC_MOUTH_ did not predict receptive language in infants in the two atypical groups (both, *p* > .10).

### MA-matched toddler comparison (this time including also a group with FXS)

#### Looking patterns–duration of fixations

**Fig 7:**
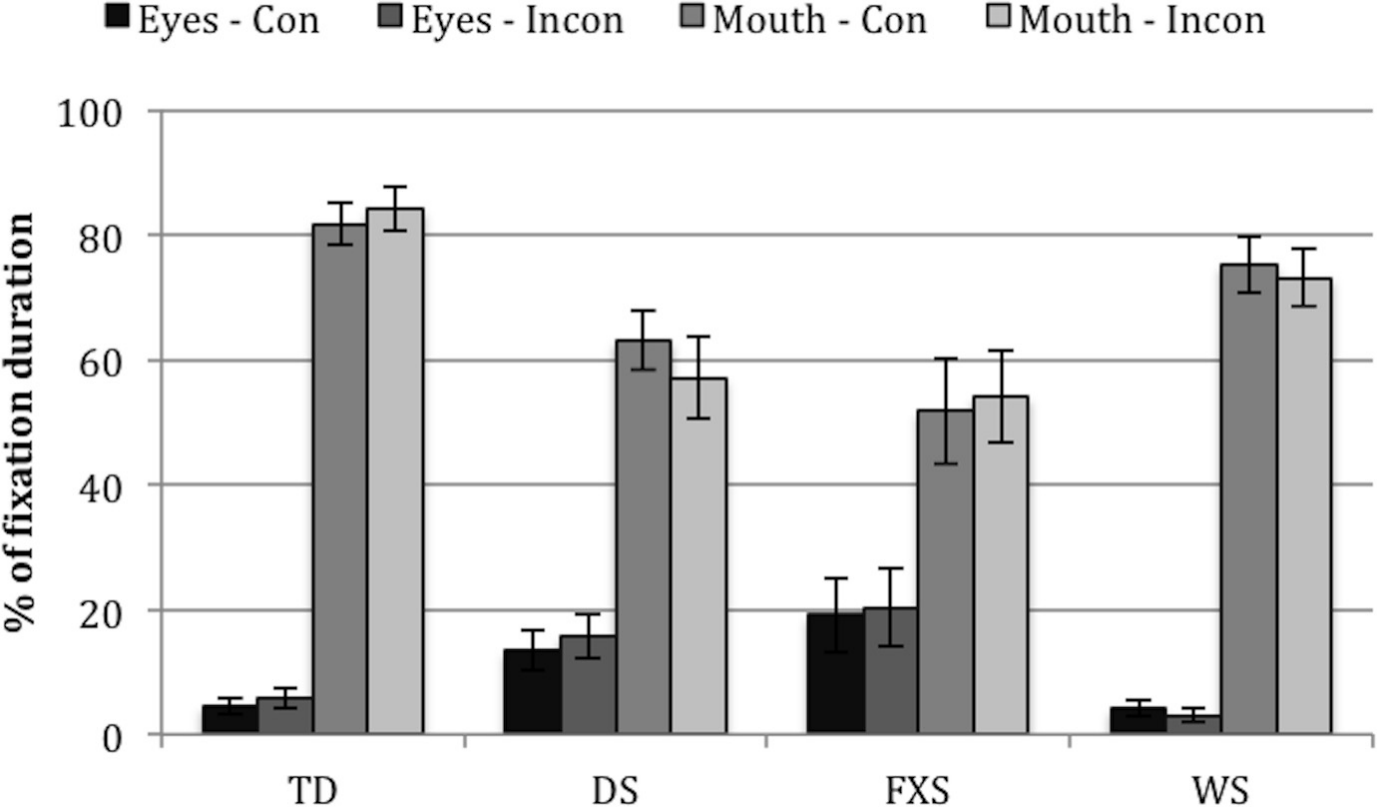
Fixation durations to eyes/mouth AOIs as a percentage of fixation durations to the face AOI, for each group (TD, DS, FXS, WS) and face (congruent, incongruent). Eyes-Con = eyes AOI in the congruent face. Eyes-Incon = eyes AOI in the incongruent face. Mouth-Con = mouth AOI in the congruent face. Mouth-Incon = mouth AOI in the incongruent face. Error bars represent one standard error of the mean.

Four fixation duration measures were analysed: *eyes in the congruent face*, *eyes in the incongruent face*, *the mouth in the congruent face*, and *the mouth AOI in the incongruent face*. These measures were re-coded as *percentages*. Fig 7 shows fixation durations to eyes/mouth AOIs as a percentage of fixation durations to the face AOI, for each group (TD, DS, FXS, WS) and face (congruent, incongruent).

Because some of the data were positively skewed while some were negatively skewed (*Z*_Skewness_ > ±2), nonparametric tests were carried out. Friedman’s ANOVAs revealed significant differences between the four measures in each of the four groups, *χ* ^2^(3) = 54.04, *p* < .001 (TD), *χ* ^2^(3) = 25.95, *p* < .001 (DS), *χ* ^2^(3) = 15.19, *p* = .002 (FXS), *χ* ^2^(3) = 52.05, *p* < .001 (WS). Wilcoxon tests were used to follow up this finding. Bonferroni corrections were applied. Therefore, all effects are reported at a .008 level of significance. TD controls fixated significantly more on the mouth (relative to the entire face) than on the eyes (relative to the entire face) (all, *p* < .001, *r* >-.76). No significant differences were found between the mouths in both faces, nor between the eyes in both faces (both, *p* > .906). The same pattern emerged in DS and WS (i.e., for all comparisons *p* < .005, *r* >-.46, except for comparisons between both mouth AOIs [both, *p* > .378] and between both eyes AOIs [both, *p* > .705]). However, this pattern may be weaker in FXS, because although the toddlers with FXS fixated less on the eyes in the congruent face than on the mouth in either face (both, *p* < .01, *r* = -.55), and less on the eyes in the incongruent face than the mouth in the congruent face (p = .008, r = -.52), the comparison between the eyes and the mouth in the incongruent face again did not survive the Bonferroni correction (*p* = .012).

Furthermore, Kruskal-Wallis tests revealed significant differences between the groups in fixation percentages to the eyes in the Congruent and Incongruent faces (*H*(3) = 13.02, *p* = .005, *H*(3) = 16.03, *p* = .001, respectively) and to the mouth in both the Congruent and Incongruent faces (*H*(3) = 16.18, *p* = .001, *H*(3) = 17.08, *p* = .001, respectively). These findings were followed up with pairwise comparisons. Bonferroni corrections were applied. Therefore, all effects are reported at a .017 level of significance. The TD controls looked significantly more at the mouth AOIs than toddlers with DS or FXS in both incongruent and congruent faces (all, *Z* > 3.00, *p* < .017). The toddlers with WS looked less at the eyes in the congruent face than toddlers with DS (*Z* = 2.89, *p* = .004) and less at the eyes in the incongruent face than both toddlers with DS or FXS (both, *Z* > 3.10, *p* < .003).

#### Looking patterns–number of fixations

**Fig 8:**
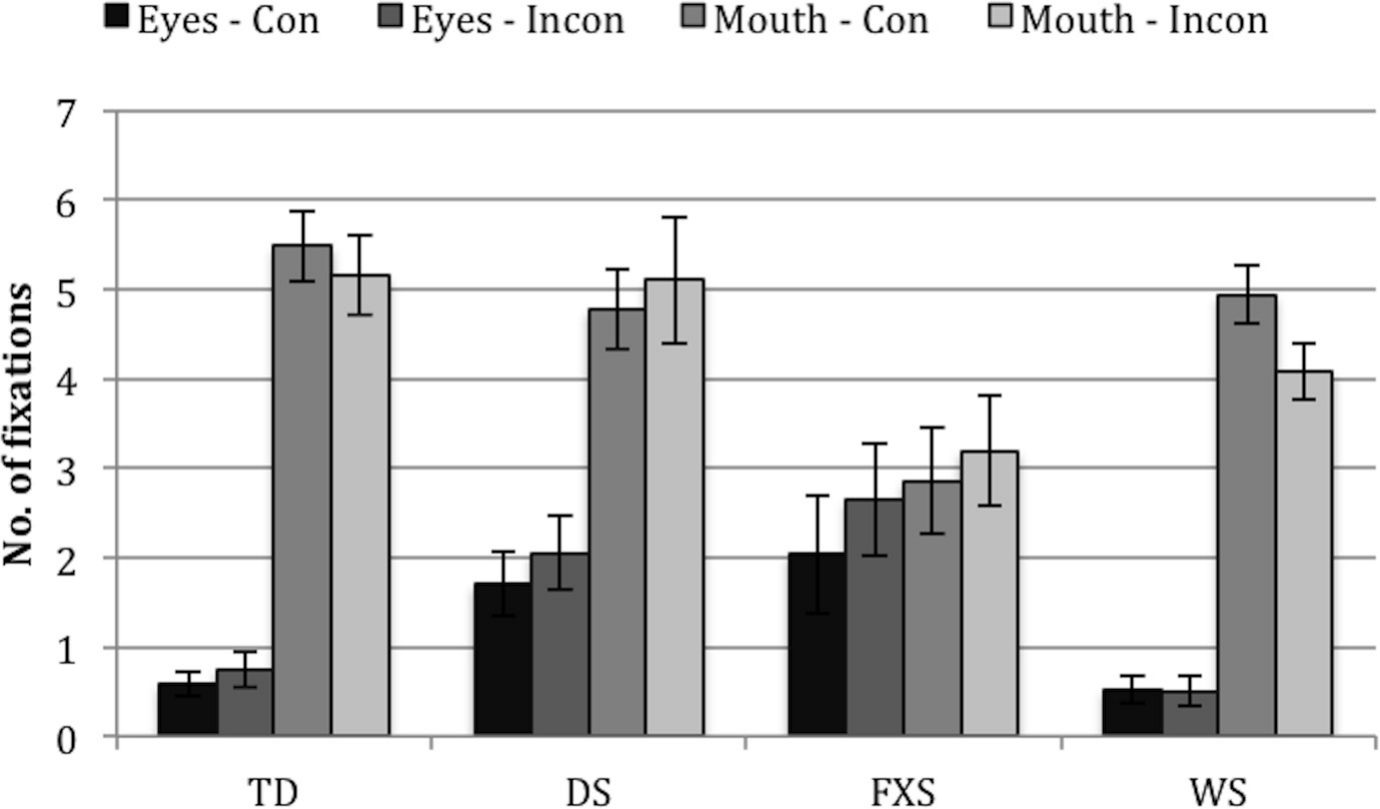
Number of fixations to eyes/mouth AOIs for each group (TD, DS, WS) and face (congruent, incongruent). Eyes-Con = eyes AOI in the congruent face. Eyes-Incon = eyes AOI in the incongruent face. Mouth-Con = mouth AOI in the congruent face. Mouth-Incon = mouth AOI in the incongruent face. Error bars represent one standard error of the mean.

We examined four ‘number of fixations’ measures: *eyes in the congruent face*, *eyes in the incongruent face*, *mouth in the congruent face*, and *mouth in the incongruent face*. Fig 8 shows number of fixations to eyes/mouth for each group (TD, DS, FXS, WS) and face (congruent, incongruent).

Because some of the data were positively skewed (even when transformed, *Z*_Skewness_ > ±2), nonparametric tests were carried out on the (untransformed) data. Friedman’s ANOVAs revealed significant differences between the four measures in some—but not all—groups, *χ* ^2^(3) = 50.88, *p* < .001 (TD), *χ* ^2^(3) = 16.86, *p* = .001 (DS), *χ* ^2^(3) = 4.15, *p* = .246 (FXS), *χ* ^2^(3) = 49.49, *p* < .001 (WS). Wilcoxon tests were then used to follow up this finding. Bonferroni corrections were applied. Therefore, all effects are reported at a .008 level of significance. TD controls made significantly more fixations to the mouths than to the eyes (all, *p* < .001, *r* >-.59). No significant differences were found between the mouths in both faces, nor between the eyes in both faces (both, *p* > .239). The same pattern emerged in WS (i.e., for all comparisons *p* < .001, *r* >-.66, except for comparisons between both mouth AOIs [*p* = .521] and between both eyes AOIs [*p* = .770]). Toddlers with DS made significantly more fixations at the mouth in the congruent face than at the eyes in either face (both, *p* < .005, *r* >-.53), but no other differences survived the Bonferroni correction.

Furthermore, Kruskal-Wallis Tests revealed significant differences between the groups in number of fixations to the eyes in both the Congruent and Incongruent face (*H*(3) = 13.88, *p* = .003, *H*(3) = 18.19, *p* < .001, respectively), and to the mouth in the Congruent, but not the Incongruent, face (*H*(3) = 11.75, *p* = .008, *H*(3) = 7.56, *p* = .056, respectively). These findings were then followed up with pairwise comparisons. Bonferroni corrections were applied. Therefore, all effects are reported at a .017 level of significance. The TD controls made significantly more fixations at the mouth in the congruent face than the toddlers with FXS, *Z* = 3.37, *p* = .001. The toddlers with DS made more fixations to the eyes than the toddlers with WS and TD controls (both, *Z* > 2.73, *p* < .007) in the congruent face. The toddlers with DS also made more fixations to the eyes than toddlers with WS in the incongruent face (*Z* = 3.15, *p* = .002). Finally toddlers with FXS made more fixations to the eyes in the incongruent face than toddlers with WS and TD controls (both, *Z* > 2.81, *p* < .006).

#### Predicting language

As with the infant groups, neither fixation duration nor number of fixations (i.e., to the mouth in the incongruent face) predicted receptive/expressive language in the three atypically developing toddler groups (all, *p* = ns).

Nevertheless, because these groups did not focus their attention on the mouth AOIs to the same extent as the TD controls, and because they made a lot of fixations to the eyes, we decided to see whether fixation to the *eyes* predicted language ability.

Fixation duration to the eyes in the incongruent face (as a percentage of fixation durations to the entire incongruent face) predicted receptive language in the FXS and WS groups ((*B* = -2.51, *SE B* = 0.72, β = -.82, *p* = .013 [which explains 67% of the variance]), *B* = 6.89, *SE B* = 3.16, β = .47, *p* = .044 [which explains 22% of the variance], respectively). Fixation duration to the eyes in the incongruent face also predicted expressive language in WS (*B* = 6.81, *SE B* = 2.20, β = .61, *p* = .007 [which explains 38% of the variance]).).

Furthermore, number of fixations to the eyes in the incongruent face predicted receptive language in the FXS group (*B* = -23.03, *SE B* = 6.48, β = -.80, *p* = .009 [which explains 64% of the variance]) and expressive language in the WS group (*B* = 35.96, *SE B* = 13.51, β = .54, *p* = .016 [which explains 29% of the variance]).

Again, to explore whether it was important which face (congruent, incongruent) the toddlers focused on, we ran regression analyses to ascertain whether FC_EYES_ or FC_MOUTH_ predict receptive language.

#### Eyes

**Fig 9:**
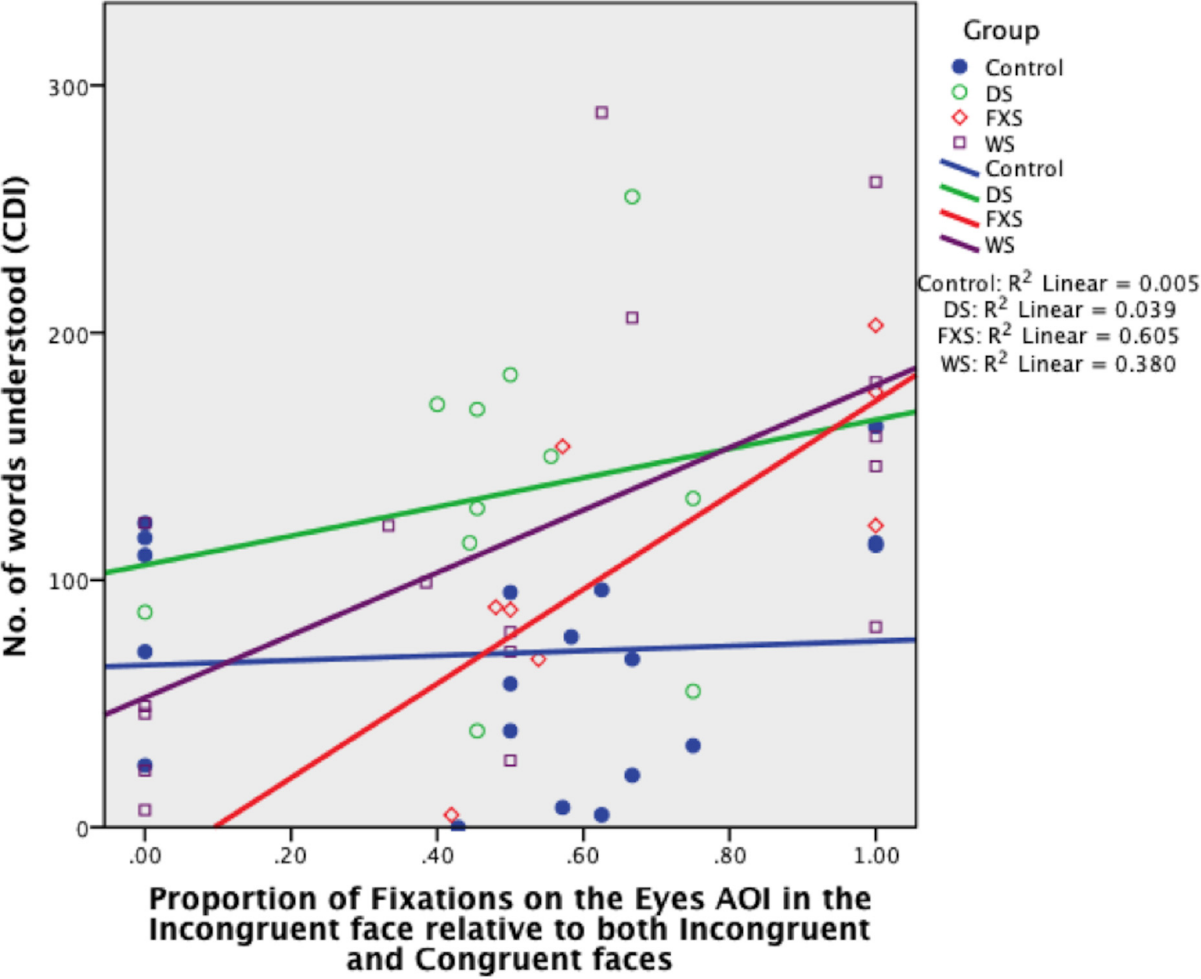
Proportion of fixations on the eyes of the Incongruent face relative to the eyes of both Incongruent and Congruent faces, organised by Group (TD control, DS, FXS, WS).

**Fig 10:**
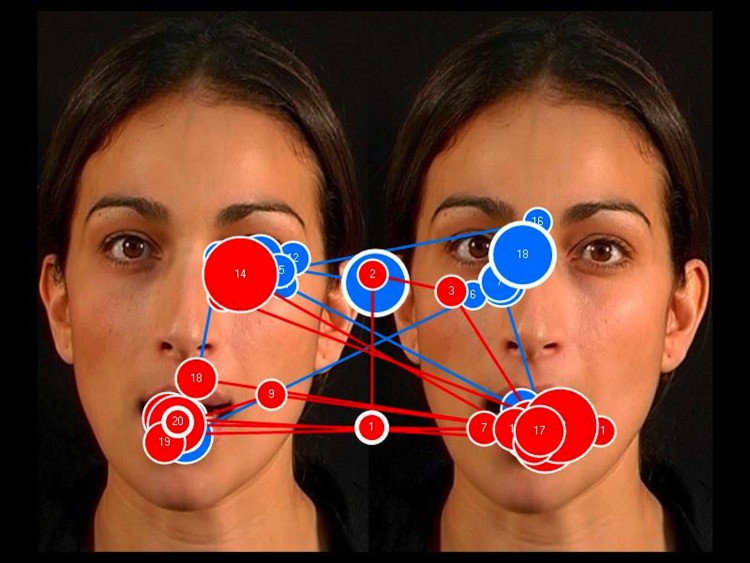
The face-scanning patterns of two toddlers with Down syndrome (DS): the blue path represents the scanning pattern of a child with a receptive vocabulary of 115 words; the red path represents the scanning pattern of a child with a receptive vocabulary of 55 words. No relationship was found in DS between face scanning and language ability. The incongruent face is the one on the left.

**Fig 11:**
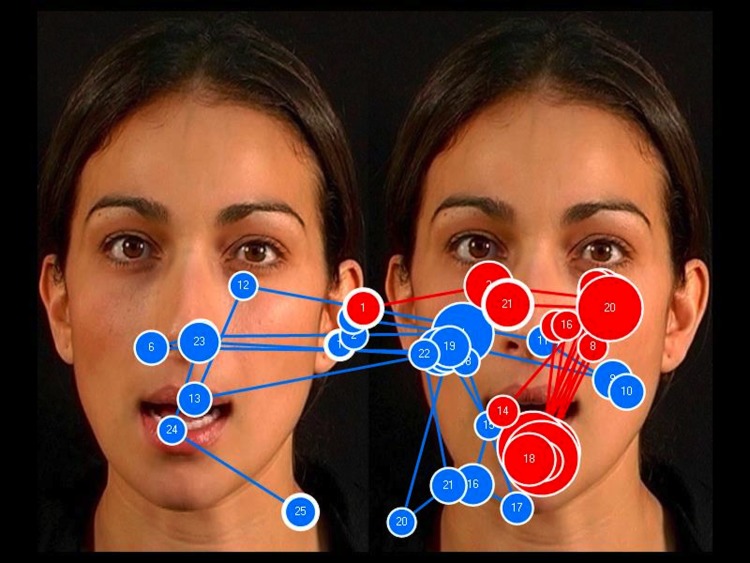
The face-scanning patterns of two toddlers with fragile X syndrome (FXS): the blue path represents the scanning pattern of a child with a receptive vocabulary of 176 words; the red path represents the scanning pattern of a child with a receptive vocabulary of 5 words. Toddlers with FXS who focused more on the eyes in the incongruent display had a larger vocabulary than those who focused more on the eyes in the congruent display. The eyes in the incongruent face are on the left.

**Fig 12:**
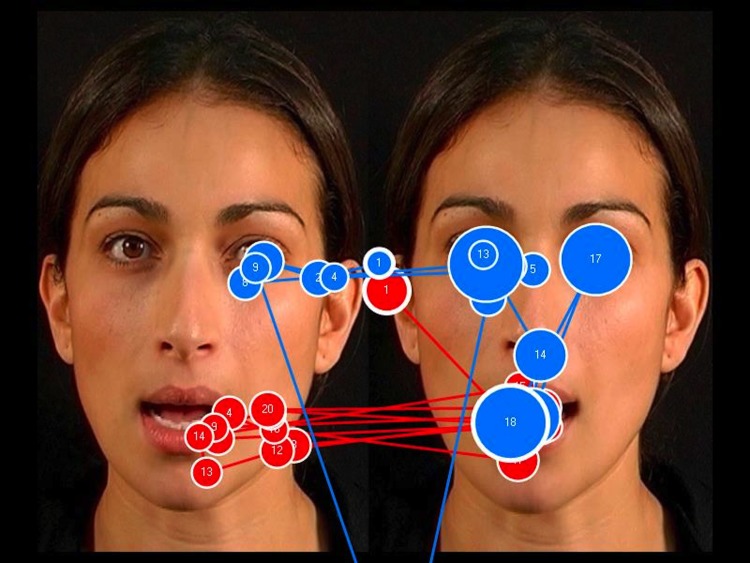
The face-scanning patterns of two toddlers with Williams syndrome: the blue path represents the scanning pattern of a child with a receptive vocabulary of 246 words; the red path represents the scanning pattern of a child with a receptive vocabulary of 23 words. Toddlers with WS who focused more on the eyes in the incongruent display had a larger vocabulary than those who focused more on the eyes in the congruent display. The eyes in the incongruent face are on the left.

FC_EYES_ was a significant predictor of receptive language and accounted for 61% of its variability in the FXS group, *B* = 190.49, *SE B* = 62.85, *β* = .78 (*R* ^2^ = .61, *p* = .023), and 38% in the WS group, *B* = 126.37, *SE B* = 41.69, *β* = .62 (*R* ^2^ = .38, *p* = .008) (see Fig 9). FC_EYE_ did not predict receptive language in the TD control or DS groups, *B* = 9.72, *SE B* = 32.98, *β* = .07 (*p* = .772), *B* = 58.73, *SE B* = 97.42, *β* = .20 (*p* = .562), respectively. This suggests that the more fixations to the eyes in the Incongruent face, relative to the eyes in both faces, the greater is the child’s receptive vocabulary (for every .1, the toddler with FXS understands an extra 19 words on average, while the toddler with WS understands an extra 13 words on average [see Figs 10, 11 and 12 for example of gaze patterns in participants with DS, FXS, and WS]).

#### Mouth

**Fig 13:**
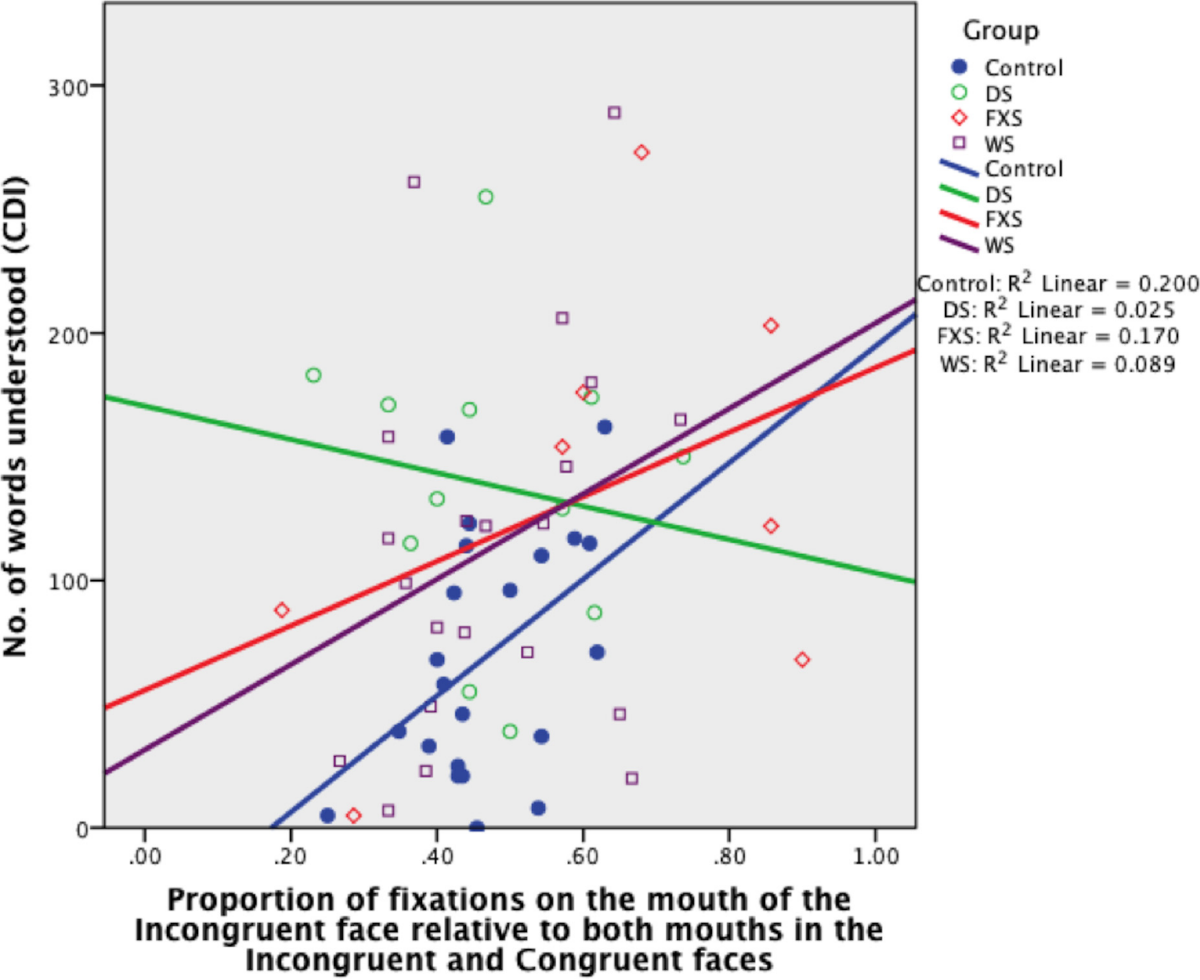
Proportion of fixations on the mouth of the Incongruent face relative to the mouths of both Incongruent and Congruent faces, organised by Group (TD control, DS, FXS, WS).

FC_MOUTH_ was a significant predictor of receptive language in toddlers and accounted for 20% of its variability in the TD control group, *B* = 235.23, *SE B* = 105.04, *β* = .45 (*R* ^2^ = .20, *p* = .037; Fig 13). This indicates that the more fixations to the mouth in the Incongruent face, relative to the mouths in both faces, the greater is the child’s receptive vocabulary (for every .1, the TD child understands an extra 24 words on average). FC_MOUTH_ did not predict receptive language in any of the atypical groups (all, *p* > .10).

### Why does greater attention to the eyes positively correlate with language ability in FXS and WS, but not in DS or TD?

One reason why looking at the eyes is correlated with language ability in children with FXS and WS is that language ability is correlated with looking at the face more generally in these children. To test this hypothesis, we carried out further analyses to ascertain whether any of the following predicts language ability: (1) proportion of target looking; i.e., fixation duration to the incongruent face as a proportion of fixation duration to both incongruent and congruent faces), (2) *total looking time* at both the incongruent and congruent faces, (3) *total number of fixations* to both incongruent and congruent faces. We would expect proportion of target looking (which demonstrates an ability to discriminate between stimuli) to be correlated with greater receptive/expressive vocabulary. We would also expect longer looking time or more fixations to the faces to correlate with language ability.

All data (for both CA- and MA-comparisons) were normally distributed (Kolmogorov-Smirnov test: all, *p* > .05; *Z*_skewness_ < 2). For the CA-comparison, while proportion of target looking did not significantly differ across groups (*F*_2,54_ = 0.22, *p* = .808), total duration and total number of fixations significantly varied across groups (*F*_2,55_ = 12.29, *p* < .001, *F*_2,53_ = 24.53, *p* < .001, respectively). Contrary to expectations, infants with WS looked significantly less at the faces than infants with DS and TD controls (both, *p* < .005, Bonferroni corrected). Perhaps participants with WS are more likely to turn away from the computer face stimuli in favour of their caregiver’s face. Infants with DS made significantly more fixations than infants with WS and TD controls (both, *p* < .001, Bonferroni corrected). Total duration was a significant predictor of expressive language in DS (*B* = -0.48, *SE B* = 0.18, β = -.68, *p* = .030 [which explains 47% of the variance]), but importantly, no variables predicted either receptive or expressive language in any of the other groups (all, *p* = ns).

For the MA-comparison, proportion of target looking did not significantly differ across groups (*p* = ns). However, total duration and total number of fixations to both faces did differ across groups, *F*_3,76_ = 13.58, *p* < .001, *F*_3,78_ = 7.51, *p* < .001, respectively. Post hoc tests revealed that the toddlers with DS looked longer at the faces and made more fixations than the other groups (all, *p* < .005, Bonferroni-corrected). Crucially, none of the variables predicted receptive or expressive language in any of the groups (all, *p* = ns).

In other words, general looking at faces was not predictive of language ability in FXS or WS, but was in the DS group.

## Discussion

The aim of the present study was to investigate the face scanning patterns of infants and toddlers with different neurodevelopmental disorders, and to ascertain whether such patterns relate to language ability in any of the groups. Specifically, we argued that if gaze to the mouth plays an important role in extracting visual information that facilitates understanding of unfamiliar or confusing (auditory) speech, then children who focus their attention towards the mouth of an incongruent talking face would have relatively larger vocabularies than those who direct their attention elsewhere. This is indeed what we found for the TD controls. The number of times and duration a TD child looked at the mouth region of a speaking face that was incongruent with the audible sound, the more likely it was that s/he had a relatively large receptive or expressive vocabulary. This makes sense, because watching lip movements influences auditory perception (e.g., phoneme discrimination [14–18, 60]), and auditory perception is an important precursor to learning spoken language [61–62].

By contrast, no relationship was found between gaze to the mouth and language ability in any of the three atypically developing groups. However, the number of times a toddler with FXS or WS looked at the *eyes* of a speaking face that was incongruent with the audible sound, the more likely it was that s/he had a relatively large receptive or expressive vocabulary. This is an unexpected finding–in part because children with FXS present with social anxiety and shyness that often result in aversion to eye contact [42]. It is not surprising that the participants with FXS did look at the eyes AOI, though. Unlike children with autism spectrum disorder, individuals with FXS are not often *uninterested* in social interactions; they avert their gaze only because they are initially shy and anxious when meeting strangers [42]. In the present study, the toddlers with FXS had no reason to avert their gaze–because we used video clips of a speaker rather than live human interaction. We would still expect them to have developed a mouth-gaze strategy—or rely solely on auditory information—to learn language. However, our results suggest that toddlers with FXS, as well as those with WS, rely on visual input from the eyes rather than from the mouths. It is important to note that at a distance of 60 cm, the infants and toddlers in our study would be able to see the entire face in their periphery. Nevertheless, these are unexpected findings. They may reflect a lack of developing expertise (see Introduction). From as early as 2 months of age [63–64], TD infants focus on the eyes. This helps them to master joint attention, imitation, the reading of emotions, and so on. However, from around 8 months of age, a shift occurs whereby TD infants begin to look longer at the mouth [35]. We speculate that this developmental shift may not occur in FXS or WS. It is possible that those with a larger vocabulary and also FXS or WS may make a few important fixations on the mouth but turn their attention to the eyes rather than the mouth of the Incongruent face. At the very least, these data suggest that the children with FXS or WS are using a different learning strategy from the one employed by TD infants.

With respect to the DS group, fixation count did not predict receptive vocabulary, which raises the possibility that children with DS do not use precise audiovisual speech cues to bootstrap language acquisition. Indeed, overall looking at the face in general (i.e., duration of fixations) predicted expressive language in DS. Note that many of the participants with DS are being taught a signed language called Makaton. We speculate that early in development infants with DS rely more on hand movements than facial movements to learn language. Further investigation is needed to unravel this.

The strength of the current study is that it used an eye tracker to measure precisely how many times TD infants and children with different neurodevelopmental disorders fixate on two different areas of interest within the face (eyes, mouth), and that we identified interesting associations between this measure and language ability. An eye tracker is more sensitive than simple observation, and the stimuli were presented in a carefully controlled environment. However, it was beyond the scope of this paradigm to establish a causal relationship between visual scanning of faces and language development. Firstly, we studied matching of only a single syllable (/ga/). Before firm conclusions can be drawn about face scanning and language, participants need to be tested on other speech samples, including more naturalistic fluent speech. Moreover, our participants could only visually explore, not interact with, the talking faces. The children may behave differently in a more naturalistic (albeit less controlled) environment.

Nevertheless, our study provides evidence that visual scanning patterns are related to language development. We have shown that TD children who use gaze to the mouth have greater vocabularies than those who do not. Moreover, some children (namely, those with DS, FXS, and WS), known to present with language delay, failed to use gaze to the mouth when processing our stimuli. These findings raise important questions: Why do children with neurodevelopmental disorders not benefit from directing their visual attention to the mouth of a speaker? Why do the children with FXS or WS who focus on the eyes of a speaker tend to have larger vocabularies? Should interventions encourage infants with these neurodevelopmental disorders to focus more on the face in general or specific parts of the face, and should this differ across different syndromes? Is it important to know why children with neurodevelopmental disorder do not focus on the mouth like TD children? Indeed, could this knowledge help clinicians to design syndrome-specific interventions that improve language ability indirectly through methods that modify face-scanning behaviour?

In summary, our findings reveal an important association between face scanning and language ability which points to intervention strategies for language delay outside language itself. Furthermore, we demonstrated that different attentional processes underpin word learning across the different groups. TD children with a relatively large vocabulary make more fixations to the speaker’s mouth, while those with FXS or WS who also have a relatively large vocabulary make more fixations to the eyes. By contrast, in children with DS fixation count to either the eyes or the mouth failed to account for individual differences in language ability. But infants with DS who spent more time looking at the overall face had larger vocabularies than those who spent less time looking at the face. These findings indicate that different processes or strategies are likely involved in language acquisition across these neurodevelopmental disorders, at least at certain points in development, processes or strategies that future studies will need to elucidate further. It may also be useful to ascertain whether training children with neurodevelopmental disorders on precisely where to look for visual input would facilitate their understanding and learning of spoken language.
